# Case Report: Management of suprastomal granulomas and severe glottic edema after tracheostomy using suspension laryngoscopy and bronchoscopy

**DOI:** 10.3389/fmed.2025.1714601

**Published:** 2025-12-02

**Authors:** Ming Chen, Xun Li, Ke Wang, Congbin Peng, Bo Yang, Weihua Xu

**Affiliations:** 1 Department of Pulmonary and Critical Care Medicine, Tongde Hospital of Zhejiang Province, Hangzhou, Zhejiang, China; 2 College of Medicine, Jiaxing University, Jiaxing, Zhejiang, China; 3 Endoscopy Center, Tongde Hospital of Zhejiang Province, Hangzhou, Zhejiang, China; 4 Department of Anesthesiology, Tongde Hospital of Zhejiang Province, Hangzhou, Zhejiang, China; 5 Department of Pharmacy, Tongde Hospital of Zhejiang Province, Hangzhou, Zhejiang, China

**Keywords:** suspension laryngoscopy, severe glottic edema, tracheostomy, difficult laryngeal exposure, suprastomal granulomas, case report

## Abstract

This report describes the successful management of severe suprastomal granulomas and glottic edema complicating tracheostomy in a patient who underwent robot-assisted frameless stereotactic-guided removal of multiple intracranial hematomas plus thrombolytic therapy for cerebral hemorrhage. During transition from tracheostomy tube to Montgomery T-tube placement, severe glottic edema and suprastomal granulomas were identified but inadequately visualized using standard electronic bronchoscopes. To overcome this challenge, suspension laryngoscopy was employed, effectively separating the swollen vocal folds and providing a clear, open, and continuously patent working channel. Utilizing bronchoscopic guidance through this channel, the suprastomal granulomas were successfully excised with a snare. Following granuloma removal, the Montgomery T-tube was successfully inserted, replacing the tracheostomy tube and ultimately restoring normal airway ventilation. This case highlights that suspension laryngoscopy offers excellent exposure, a wide surgical view, and an unobstructed operative pathway, facilitating bronchoscope manipulation and providing an effective solution for managing severe glottic edema and suprastomal obstruction post-tracheostomy.

## Introduction

Prolonged placement of a tracheostomy tube after tracheotomy carries risks of developing complications such as granulation tissue, benign tracheal stenosis, and glottic edema (1). Suprastomal granulomas typically occur below the tracheostomy tube while some are located above the stoma (2). Depending on their location, various treatments for granulomas have been reported that include snare resection, cryotherapy (3), and thermal ablation (4). Granulomas located near or below the tracheostomy tube can be removed with a bronchoscope using the tube itself as a working channel. However, suprastomal granulomas require trans-stomal access via the mouth or nose and can present challenges for bronchoscopic intervention. In some cases, severe glottic edema co-occurring with suprastomal granulomas can further impede bronchoscopic visualization and surgical access posing a significant challenge for clinicians when performing granuloma removal surgery.

Here, we report a case involving successful removal of suprastomal granulomas and placement of a Montgomery T-tube in a patient with severe glottic edema and difficult laryngeal exposure. This was achieved using a multidisciplinary approach using suspension laryngoscopy to view and separate the swollen vocal cords. This provided an unobstructed view and working channel for bronchoscopic resection of the suprastomal granulomas.

## Case presentation

A 72-years-old male patient was admitted to our emergency intensive care unit (ICU) on February 21, 2022, due to a 2-days history of headache and impaired consciousness. He was diagnosed with intracerebral hemorrhage and had a past medical history of hypertension. Due to the severity of his condition, he underwent endotracheal intubation and emergently received robot-assisted frameless stereotactic evacuation of multiple intracerebral hematomas combined with thrombolytic therapy on February 21, 2022. A tracheostomy was performed on March 4, 2022.

During hospitalization, the patient experienced recurrent pneumonia. After anti-infective treatment, nutritional support, and bedside rehabilitation, his mental status improved and infection was controlled, with a noticeable reduction in airway secretions. To increase the likelihood of successful decannulation, a T-tube was planned as a transitional step before removing the tracheostomy tube.

**FIGURE 1:**
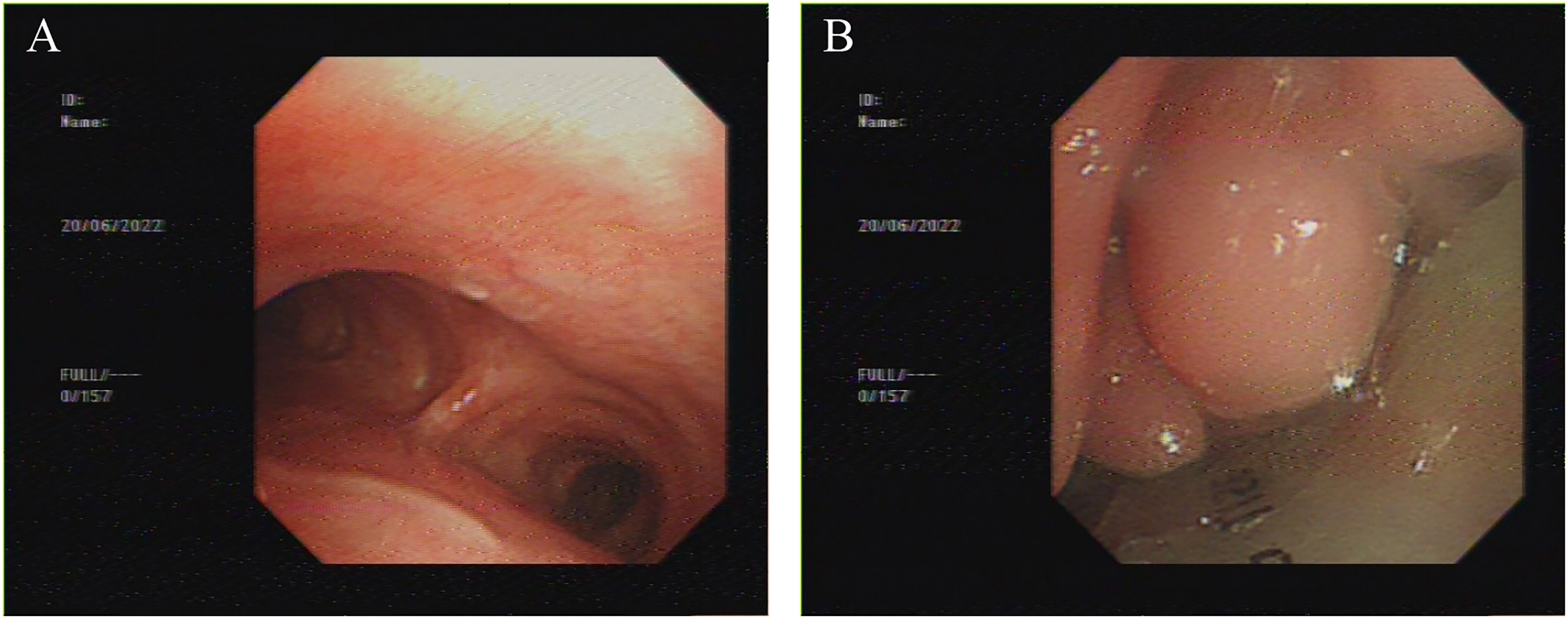
Baseline bronchoscopy (June 20, 2022). **(A)** Patent distal trachea below tracheostomy tube. **(B)** Severe supraglottic edema obscuring glottis.

On June 20, 2022, bronchoscopic T-tube placement was performed under general anesthesia. The bronchoscope was introduced through the tracheostomy tube into the distal airway, revealing no stenosis or granulation tissue (Figure 1). When attempting transglottic access via laryngeal mask, severe edema of the pharynx and glottis was noted, obscuring visualization of the vocal cords and airway. Multiple attempts were made to pass the scope into the trachea, where near-complete obstruction by granulation tissue was found above the tracheostomy balloon.

During a subsequent attempt at granulation tissue removal, repeated passage through the swollen glottis failed. Rigid bronchoscopy was also attempted but unsuccessful due to significant airway edema. Although video laryngoscopy temporarily exposed the glottis with assistance from anesthesiology, adequate surgical access could not be achieved, and the procedure was abandoned.

**FIGURE 2:**
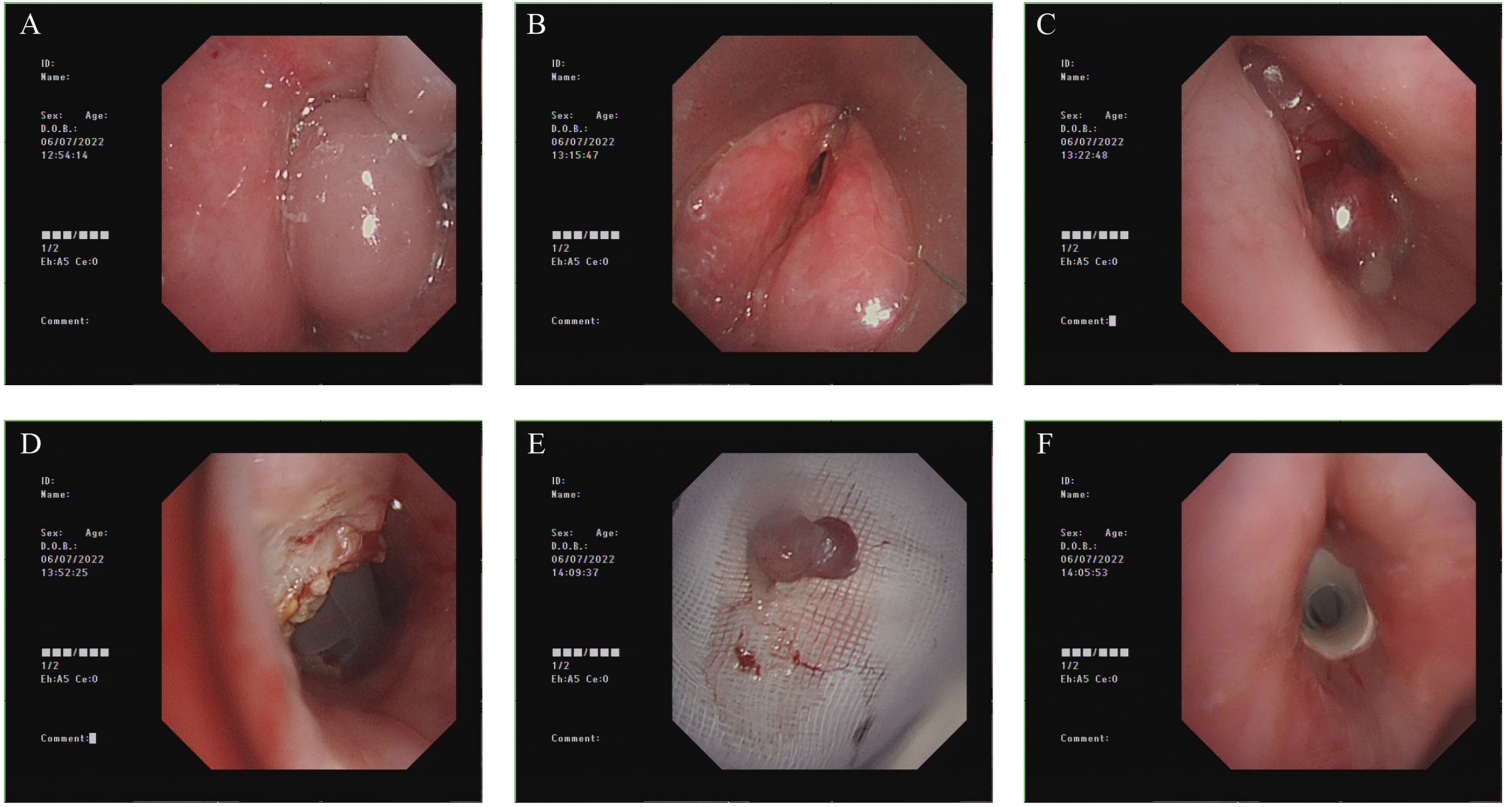
Intraoperative procedure (July 6, 2022). **(A)** Distal airway integrity. **(B)** Persistent supraglottic edema. **(C)** Suspension laryngoscopy achieving glottic exposure. **(D)** Suprastomal granuloma causing near-total obstruction. **(E)** Snare resection under bronchoscopic guidance. **(F)** T-tube placement post-resection.

After multidisciplinary discussion, we opted to use suspension laryngoscopy for glottic exposure. On July 6, 2022, bronchoscopic excision of suprastomal granulation tissue (Figure 2) with T-tube placement was performed under general anesthesia. An otolaryngologist used suspension laryngoscopy to fully expose the swollen glottis, allowing bronchoscopic access to the airway. The suprastomal granulation tissue was resected using a snare, followed by removal of the tracheostomy tube and placement of a T-tube. The procedure was completed successfully with release of the laryngoscope.

**FIGURE 3:**
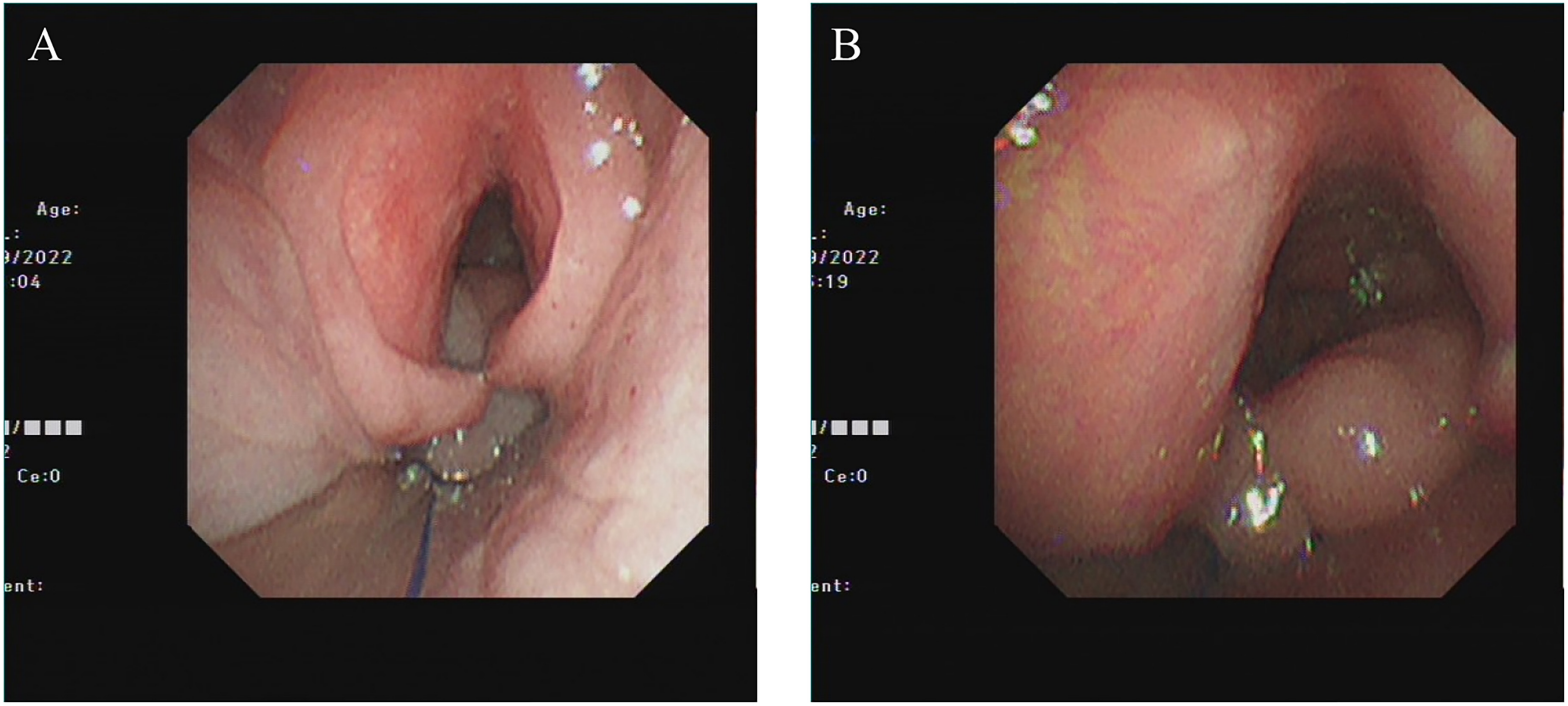
Three-months follow-up (September 12, 2022). **(A)** Marked resolution of glottic edema. **(B)** Residual subglottic granuloma.

On September 12, 2022, follow-up bronchoscopy (Figure 3) showed marked improvement in glottic edema, with only mild epiglottic swelling and a small residual granulation below the vocal cords, which did not affect ventilation. The lesion was managed conservatively with inhaled corticosteroids.

## Discussion

Prolonged placement of a tracheostomy tube after tracheotomy can result in a variety of complications, including granulomas, subglottic stenosis, vocal cord paralysis, laryngeal edema, tracheal softening, and tracheoesophageal fistula (5, 6). These complications can lead to difficulties in extubation, delayed extubation, increased healthcare expenditure, seriously impacting the quality of a patient’s life, and even endanger their life.

Granuloma formation is a common complication following tracheostomy, typically occurring around the cuff or at the tip of the tracheostomy tube. It is widely accepted that excessive pressure from the cuff exceeding the tracheal mucosal capillary perfusion pressure leads to mucosal injury, localized ischemia, and edema. Subsequent bacterial infection promotes ulceration, initiating a process of granulation, tissue hyperplasia, and repair, ultimately resulting in granuloma development. Additionally, mechanical factors such as friction from tube movement or coughing, traumatic suctioning, and repeated abrasion by the suction catheter contribute to granuloma formation at the tube tip (7). The formation of granulomas above the stoma may be related to the damage caused by prior tracheal intubation, the accumulation of secretions or chronic infection (8, 9).

Avoiding excessive cuff pressure, placing an appropriate-sized tracheostomy tube, and avoiding excessive mechanical stimulation are considered beneficial in reducing the incidence of granulomas (10). Of course, actively controlling stoma infection, corticosteroid use administering anti-reflux drugs, and mitomycin C (11) are also helpful in reducing the occurrence of granulomas. Most granulomas will resolve on their own, but larger ones often require further interventional treatment involving bronchoscopy and surgery (12).

Bronchoscopic intervention serves as the cornerstone for treating obstructive granulomas, with techniques broadly categorized by their mechanism: thermal ablation, cryoablation, and mechanical debulking. Common modalities include electrocautery, argon plasma coagulation (APC), Nd-YAG laser, and snare ligation. The electrocautery snare, a mechanical tool, is particularly effective for pedunculated lesions, allowing rapid resection. Both laser and APC function through thermal ablation, vaporizing or coagulating tissue, which makes them suitable for sessile or highly vascularized lesions. In contrast, cryotherapy induces cell necrosis via extreme cold. It offers a distinct advantage by being less destructive to the extracellular matrix, thereby preserving airway structure and promoting mucosal re-epithelialization. This property makes it valuable in cases of recurrent granulation, especially around stents, as it may help reduce the risk of restenosis. A comparative summary of these common interventional techniques is provided in Table 1.

**TABLE 1:** Comparison of interventional techniques for endobronchial granulomas.

Technique	Principle	Key advantages	Key limitations	Primary indications and notes
High-frequency electrocautery/snare	High-frequency current for cutting/coagulation.	High cutting efficiency; good for pedunculated lesions; effective hemostasis.	Risk of deep thermal injury, perforation, and fire hazard; may stimulate regrowth.	First-line for rapid debulking of pedunculated lesions. Use cutting mode, avoid coagulation.
Laser (e.g., Ho-YAG, Nd-YAG)	Light energy converted to heat for vaporization.	High precision and efficiency; Ho-YAG: shallow penetration; Nd-YAG: deep ablation and hemostasis.	Significant thermal damage (Ho-YAG); high fire risk; expensive; may promote scarring.	For rapid resection. Stop near base and use cryotherapy on the base to reduce recurrence.
Argon plasma coagulation (APC)	Non-contact coagulation via ionized argon gas.	Superficial penetration (2–3 mm); rapid treatment of large, broad-based, oozing surfaces.	Superficial effect only; no cutting ability; risk of thermal injury and “argon storm.”	For superficial, hemorrhagic, broad-based lesions. Adjunct for hemostasis.
Cryotherapy	Cell death via freezing-induced crystallization.	No thermal damage; preserves airway structure; minimal regrowth risk; cost-effective; no fire risk.	Delayed effect (1–3 days); not for critical stenosis; post-procedure edema risk.	First-line for benign granulomas. Ideal combined with thermal debulking for base treatment.

The occurrence of laryngeal edema is relatively high in patients undergoing tracheal intubation and those with long-term tracheostomy tube placement. Some studies have looked at this phenomenon statistically but, unfortunately, the underlying causes have not been analyzed (13).

Based on our limited understanding, the occurrence of laryngeal edema may be associated with obesity (BMI ≥ 25 kg/m^2^) and/or long-term inflatable cuff tracheostomy tube retention. Excessive accumulation of fat in the neck causing local compression of the cuff can lead to local ischemia and circulatory problems resulting in edema (4). In our clinical experience, the use of metal tracheostomy tubes in patients with normal body weight is associated with a relatively low incidence of laryngeal edema. However, if overlooked, laryngeal edema can lead to serious clinical outcomes, including respiratory distress or even asphyxia following decannulation. Furthermore, significant swelling in the pharyngeal and laryngeal regions may impede bronchoscopic examination, thereby complicating the evaluation and management of subsequent complications such as benign airway stenosis, tracheomalacia, and granuloma formation. While it is challenging to completely prevent laryngeal edema in this context, efforts should be made to minimize its adverse sequelae.

In the past, the use of a supportive laryngoscope focused mainly on treatment of vocal cord lesions and benign tumors such as vocal polyps, hemangiomas, papillomas, and arytenoid cysts in the pharynx and larynx (14, 15). With increased interdisciplinary cooperation, the use of a supportive laryngoscope needs no longer to be limited to otolaryngological diseases. Several researchers have used supportive laryngoscopes for tracheotomy (16), removal of tracheal stents, insertion of airway stents, and airway dilation (17).

A number of studies have shown that in approximately 8% of patients use of traditional laryngoscopes or rigid bronchoscopes does not permit good glottic exposure, even when performed by technically experienced physicians (18). In the present study, we report a similar type of situation where severe swelling of the glottic area, obesity, and a short neck contributed to this problem. Supportive laryngoscopes can be useful in this situation by providing continuous and effective traction allowing exposure of the swollen glottis and permitting the bronchoscope to freely and easily enter the airway. In addition, maintenance of a continuously patent surgical channel that frees both hands allows the operating physician to focus on the procedure.

## Conclusion

In summary, our team’s groundbreaking application of a supportive laryngoscope for treating patients with severe glottic edema after tracheotomy provides excellent exposure, a wide surgical view, continuously patent surgical channels, and ease of bronchoscope operation. These advantages can create optimal conditions for subsequent treatment of various airway problems. The cost-effectiveness and wide availability of supportive laryngoscopes can even replace rigid bronchoscopes to a certain extent, which may be beneficial for some medical institutions with limited facilities and equipment.

## Data Availability

The original contributions presented in this study are included in this article/supplementary material, further inquiries can be directed to the corresponding authors.
